# Connecting for Success: The Role of Networking in Medical Education

**DOI:** 10.7759/cureus.74643

**Published:** 2024-11-28

**Authors:** Pinaki Wani

**Affiliations:** 1 Physiology, All India Institute of Medical Sciences, Raebareli, Raebareli, IND

**Keywords:** career advancement, collaboration, medical education, networking, professional development, research opportunities

## Abstract

Networking is a critical component of professional development in medical education, involving the establishment and maintenance of relationships that facilitate the exchange of information, resources, and opportunities. Defined as the process of creating and nurturing connections with peers, mentors, and collaborators, networking is essential for advancing research, enhancing career development, and improving clinical practice. This review explores the multifaceted role of networking in academia, emphasizing its importance for medical professionals who rely on collaborative efforts to drive innovation and improve patient care. Key benefits of strategic networking include access to interdisciplinary research opportunities, enhanced knowledge sharing, career advancement, and resource acquisition. However, networking in medical education is not without challenges. Time commitment and burnout, superficial relationships, exclusivity, ethical concerns, and professional jealousy are potential drawbacks that can hinder the effectiveness of networking efforts. Solutions to these challenges include balancing networking with other responsibilities, focusing on quality over quantity, ensuring inclusivity, and maintaining ethical standards in professional relationships. The review also provides practical tips for effective networking, such as engaging in formal and informal networking activities, leveraging technology, and building a professional online presence. By strategically participating in networking activities and overcoming common challenges, medical professionals can significantly enhance their academic growth, career opportunities, and contributions to the field. The overarching message is that investing in and nurturing professional networks is crucial for sustained success and advancement in medical education and research.

## Introduction and background

Definition of networking

Networking is the process of establishing and nurturing professional relationships to exchange information, resources, and opportunities. It involves creating connections with individuals or groups who can provide support, share knowledge, or collaborate on various projects. In academic settings, networking typically includes interactions at conferences, workshops, seminars, and through professional societies or online platforms [1,2]. Networking in academia plays a critical role in advancing scholarly work and career development. It facilitates the exchange of ideas, collaboration on research projects, and access to funding opportunities. Academic networking can lead to fruitful partnerships, enhance professional visibility, and provide access to valuable resources and expertise. A study by Ismail and Rasdi highlighted the importance of networking among women academicians in Malaysia [3]. For medical professionals, networking is particularly crucial due to the collaborative nature of medical research and clinical practice. Networking allows for the exchange of clinical insights, research findings, and innovations that can enhance patient care and advance medical knowledge. It also provides opportunities for professional development, such as mentorship and leadership roles within medical societies [4,5]. For example, a medical educator who actively participates in professional societies and engages with peers at international conferences may gain access to innovative teaching methods and research collaborations. This can result in improved educational programs and enhanced patient care practices. For instance, networking with colleagues from different institutions can lead to the development of a collaborative research study that investigates new treatment protocols, ultimately benefiting patient outcomes and advancing the field [6]. Hence, networking in the medical profession provides essential benefits for academic growth, including access to collaborative opportunities, knowledge exchange, and professional development. Engaging with peers and experts through various networking channels can significantly impact career advancement and contribute to the overall progress of medical science and education.

## Review

Benefits of strategic professional networking in medical education

Collaboration and Research Opportunities

Interdisciplinary research: Networking allows medical professionals to collaborate across various disciplines, enhancing the scope and depth of research. The Framingham Heart Study exemplifies successful interdisciplinary collaboration. Researchers from multiple fields have worked together to understand cardiovascular disease, leading to numerous breakthroughs in prevention and treatment [7].

Joint projects and grants: Networking facilitates partnerships that lead to joint research projects and grant applications. Collaborative projects often have a higher chance of receiving funding due to the combined expertise and resources of the team. The National Institutes of Health (NIH) often funds collaborative research projects, such as those involving multiple institutions working together to study complex diseases like cancer or diabetes [8].

Knowledge Sharing and Skill Enhancement

Exchange of ideas and best practices: Networking enables the exchange of ideas and best practices, leading to improved methodologies and techniques in medical practice and education. An example is the annual American Medical Association (AMA) meetings provide a platform for professionals to share innovative practices and emerging research findings, which can then be implemented in their institutions [9].

Learning from peers and mentors: Networking provides opportunities to learn from experienced peers and mentors, which can accelerate personal and professional growth. Many medical schools and institutions offer mentorship programs where junior faculty members can receive guidance from senior colleagues, enhancing their research and teaching skills [10].

Career Advancement

Visibility and recognition: Networking helps increase visibility and recognition within the medical community, leading to career advancement opportunities. Active participation in conferences and professional societies can lead to invitations to speak, publish, or join editorial boards, enhancing one's professional reputation [11].

Mentorship and professional development: Through networking, professionals can access mentors who provide valuable career advice, support, and opportunities for professional development [12].

Access to Resources

Funding opportunities: Networking helps professionals discover funding opportunities for research and development projects, often through connections with grant organizations and funding bodies. The National Science Foundation (NSF) offers various grants for medical research, and networking can provide insights into upcoming funding calls and application tips [13].

Institutional support and shared resources: Networking can lead to access to institutional resources and support, such as shared research facilities, technology, and collaborative platforms. For example, the European Research Council (ERC) funds collaborative projects that utilize shared resources across institutions, promoting cutting-edge research in various medical fields [14].

Methods and strategies for effective networking in medical education

Formal Networking

Attending conferences and workshops: Conferences and workshops provide opportunities for medical professionals to engage with peers, learn about the latest research, and participate in hands-on sessions. These events are crucial for staying updated on advancements and for meeting leading experts in the field. For example, the American College of Physicians (ACP) Annual Meeting is a prime example where attendees can network with colleagues, participate in educational sessions, and explore new research findings [15].

Participating in professional societies and associations: Joining professional societies such as the American Society for Cell Biology (ASCB) and the American Society for Biochemistry and Molecular Biology (ASBMB) provides access to exclusive networking events and publications for advancement in career [16].

Informal Networking

Building relationships with colleagues: Developing relationships with colleagues in your institution or field can lead to collaborative opportunities and provide support for professional development. These informal connections often lead to meaningful partnerships and shared projects. A study by Ward et al. highlighted how informal networking among colleagues facilitated collaborative research and improved job satisfaction among medical professionals [17].

Using social media and online platforms: Social media platforms like LinkedIn, X (formerly known as Twitter), and ResearchGate provide spaces for professionals to share research, engage in discussions, and connect with others in their field. These platforms can facilitate networking beyond geographic constraints. For example, Twitter-based networks such as #MedEdChat help medical educators and researchers share resources, discuss educational strategies and collaborate on projects [18].

Mentorship Programs

Seeking mentorship and becoming a mentor: Mentorship provides guidance and support for career development. Being a mentor not only helps others but also strengthens professional relationships and enhances one's own career growth [19].

Establishing long-term professional relationships: Cultivating long-term relationships with mentors and colleagues can result in enduring collaboration and support throughout one's career. These connections often offer continuous professional guidance, collaborative opportunities, and pathways for career advancement. For instance, mentorship relationships developed through programs like the NIH Career Development Awards have demonstrated a significant impact on career growth and research productivity [20].

Cross-Institutional Collaborations

The following methods and strategies for effective networking provide a comprehensive approach to fostering professional growth and academic development among medical professionals, helping medical professionals enhance their careers, contribute to advancements in their field, and improve medical education.

Engaging in multi-center research projects: Collaborating on multi-center research projects allows for pooling resources, data, and expertise from different institutions, leading to more comprehensive research outcomes and increased publication opportunities. A multi-national clinical trial conducted by the Global Cardiovascular Research Network (GCRN) involved multiple institutions and demonstrated the benefits of cross-institutional collaboration for advancing cardiovascular research [21].

Developing cross-institutional teaching initiatives: Collaborating with other institutions to develop and deliver educational programs can enhance the quality of medical education and provide diverse perspectives and resources. For example, the MedEdPORTAL collaboration between multiple medical schools developed a shared repository of educational materials and best practices, improving the quality of medical education across institutions [22].

Potential challenges and drawbacks of networking in medical education

Time Commitment and Burnout

Networking often requires significant time investment, including attending conferences, meetings, and other professional events. This added burden can contribute to burnout for medical professionals, especially when combined with the demands of clinical work, teaching, and research. For instance, a study found that the time demands of academic networking can contribute to professional burnout among medical faculty, particularly when they already face high clinical workloads [23].

Superficial Relationships and Networking Fatigue

Networking can sometimes lead to the formation of superficial connections that lack depth. Additionally, constant networking efforts can lead to networking fatigue, where professionals feel overwhelmed by maintaining numerous weak relationships. An investigation into academic networking revealed that while networking can expand professional circles, it often results in many low-value connections that do not lead to meaningful collaborations or professional growth [24].

Exclusivity and Inequality in Access

Not all medical professionals have equal access to networking opportunities, which can create disparities based on geographic location, institutional support, and financial resources. This exclusivity can hinder the professional growth of those who are less connected. A study highlighted that networking opportunities are often concentrated in major academic centers, leaving professionals in less prominent institutions at a disadvantage [25].

Ethical Concerns

Networking can sometimes lead to ethical issues, such as conflicts of interest or biased decision-making. The influence of personal relationships may affect academic decisions related to funding, promotions, and publications. Research has shown that strong professional relationships can sometimes lead to conflicts of interest, where personal connections might influence decisions on grant allocations or publication opportunities [26].

Professional Jealousy and Competition

Networking can exacerbate feelings of jealousy and competition among professionals, especially in competitive academic environments where individuals are vying for similar opportunities. A study on academic medicine revealed that intense networking can heighten professional rivalries and jealousy, which can impact collaboration and the overall work environment [27].

Overcoming networking challenges in the medical profession

The following strategies illustrate how medical professionals can effectively navigate the challenges of networking, ensuring that their efforts contribute positively to their academic and professional growth.

Balancing Networking With Other Responsibilities

Medical professionals often face time constraints due to their clinical duties, teaching responsibilities, and research commitments. Balancing networking activities with these responsibilities can be challenging. These issues can be resolved with the following strategies:

Effective time management: Prioritize networking activities that align closely with professional goals and integrate them into your schedule without compromising clinical and academic duties.

Strategic participation: Choose networking events and opportunities that offer the most value for your specific career goals. This may include selective participation in high-impact conferences or focused professional groups.

Delegation: Engage in networking activities that can be managed alongside daily responsibilities, such as virtual meetings or online professional forums [28].

Focusing on Quality Over Quantity

Networking often emphasizes the quantity of connections rather than the quality of interactions, which can lead to superficial relationships that offer little professional value. Recommendations for resolving such issues are as follows:

Targeted networking: Focus on building deeper relationships with key individuals who can offer meaningful professional insights and opportunities. Engage in substantive discussions and collaborations rather than simply increasing the number of contacts.

Follow-up: Prioritize follow-up with connections that show potential for collaboration or mentorship, ensuring that relationships are nurtured and developed over time [29].

Ensuring Inclusivity in Networking Opportunities

Networking opportunities can sometimes be exclusive, creating barriers for individuals from underrepresented backgrounds or those with limited access to resources. Possible avenues to alleviate this lack could involve the following strategies:

Inclusive networking events: Promote and participate in networking events that focus on inclusivity and diversity, ensuring that opportunities are accessible to a broad range of professionals.

Mentorship and support: Actively support and mentor individuals from diverse backgrounds, helping them navigate networking opportunities and build their professional networks [30].

Maintaining Ethical Standards in Professional Relationships

Networking can sometimes lead to ethical dilemmas, such as conflicts of interest or biased decision-making influenced by personal connections.

Transparency: Maintain transparency in professional relationships and decision-making processes, ensuring that personal connections do not unduly influence academic or clinical outcomes.

Ethical guidelines: Adhere to institutional and professional ethical guidelines that govern interactions and relationships, ensuring that all networking activities are conducted with integrity and professionalism [31].

Case studies

The following examples demonstrate how effective networking, collaborative initiatives and mentorship can lead to significant advancements in research, teaching, and career development within the medical profession:

Successful Networking Leading to Major Research Breakthroughs

The Human Genome Project: This is a landmark example of successful networking leading to major research breakthroughs. This international research initiative aimed to map and understand all the genes of the human species. The project required extensive collaboration among scientists, institutions, and countries. Networking facilitated the sharing of data, resources, and expertise, which was crucial for its success [32].

Collaborative Teaching Initiatives Enhancing Medical Education

Problem-based learning (PBL): This model is a collaborative teaching initiative that has enhanced medical education worldwide. PBL involves students working in groups to solve clinical problems, promoting active learning and critical thinking. The implementation of PBL in medical schools requires collaboration among educators to develop effective curricula and teaching strategies. This networking among educators led to improved teaching methods and student outcomes [33].

Mentorship Leading to Career Advancement

The role of mentorship in academic medicine: Mentorship is crucial for career advancement in academic medicine. A well-documented example is the mentoring relationship between Dr. Harold Varmus and Dr. J. Michael Bishop, who both won the Nobel Prize in Physiology or Medicine in 1989 for their discoveries related to cancer research. Their mentorship and collaborative work significantly advanced their careers and contributed to major breakthroughs in cancer research [34,35].

Practical tips for networking in medical education

The following practical tips for networking in medical education not only help build and maintain professional relationships but also enhance academic growth and career development for medical professionals:

Effective Communication and Active Listening

Clear and concise communication is essential for building strong professional relationships. Medical professionals should aim to articulate their research interests, clinical expertise, and academic goals engagingly and understandably to others in their field.

Active listening: This involves giving full attention to the speaker, understanding their message, and responding thoughtfully. It demonstrates respect and fosters meaningful connections. In medical education, active listening can enhance collaborative efforts, improve mentoring relationships, and facilitate successful interactions with peers and mentors [36].

Follow-up Strategies

After initial meetings or networking events, it’s crucial to follow up with the contacts made. This can include sending a thank-you note, referencing a specific part of your conversation, or proposing a follow-up meeting. Consistent and thoughtful follow-up helps to solidify new connections and keep the dialogue open [37]. The following practical tips for networking in medical education not only help build and maintain professional relationships but also enhance academic growth and career development for medical professionals:

Leveraging Technology for Networking

Digital platforms, such as LinkedIn, ResearchGate, and Twitter, provide opportunities to connect with other professionals, share research findings, and stay updated on industry trends. Using these tools effectively can expand one's network beyond traditional face-to-face interactions [38].

Building a professional online presence: Establishing a professional online presence through platforms like LinkedIn, personal academic websites, and professional blogs is essential for visibility. This includes regularly updating profiles, showcasing achievements, and engaging with relevant content to attract opportunities and demonstrate expertise [39].

## Conclusions

In conclusion, networking is a vital tool for advancing academic growth in medical education, fostering collaboration, enhancing career opportunities, and providing access to invaluable resources. By actively participating in networking activities, medical professionals can build meaningful relationships, stay updated with the latest advancements, and contribute to a supportive academic community. The take-home message is clear: invest in building and nurturing your professional network to unlock new opportunities and drive sustained success in your academic and professional journey.
